# Vocal cord paralysis: anatomy, imaging and pathology

**DOI:** 10.1007/s13244-014-0364-y

**Published:** 2014-10-15

**Authors:** J. W. Dankbaar, F. A. Pameijer

**Affiliations:** Department of Radiology (HP E01.132), University Medical Center Utrecht, PO Box 85500, Utrecht, 3508 GA The Netherlands

**Keywords:** Vocal cord paralysis, Vocal cord anatomy, Vagal nerves, Laryngeal nerves, Imaging

## Abstract

Vocal cord paralysis (VCP) can be caused by any process that interferes with the normal function of the vagal nerves or recurrent laryngeal nerves. It may be a first sign of extensive and severe pathology. Radiologists must therefore be able to recognise the imaging findings of VCP and know the course of the vagal and recurrent laryngeal nerves. This review focuses on the anatomy and imaging evaluation of these nerves and thereby the possible sites for pathology causing VCP. The imaging characteristics and imaging mimics of VCP are discussed and cases from daily practice illustrating causes of VCP are presented.

• *Vocal cord paralysis may be the first presentation of severe pathology.*

• *Radiologists must be aware of imaging characteristics and mimics of vocal cord paralysis.*

• *Lesions along the vagal nerves and recurrent laryngeal nerves can cause vocal cord paralysis.*

## Introduction

The vocal cords play a crucial role in phonation. The muscles that are responsible for vocal cord movement are mainly innervated by the recurrent laryngeal nerves. The recurrent laryngeal nerves are branches of the vagal nerves. Vocal cord paralysis (VCP) can therefore be caused by any lesion along the course of the vagal nerves above the branching of the recurrent laryngeal nerves or of the recurrent laryngeal nerves itself. An offending lesion located in the brainstem or the skull base usually results in multiple cranial nerve deficits because at this level the vagal nerve is intimately related to other cranial nerves. Pathology involving the recurrent laryngeal nerves and/or the extracranial vagal nerves frequently results in isolated laryngeal symptoms. VCP most frequently affects one side but can be bilateral. Due to long anatomical course of the vagal and recurrent laryngeal nerves, there are many disease processes that can cause VCP. Surgery, malignancy, trauma, infection and inflammation can all result in VCP. A review of more than 800 patients showed that iatrogenic injury by mediastinal and neck surgery is the most important cause of VCP [1]. Around 40 % of unilateral VCP and 50 % of bilateral VCP is caused by surgical injury. Bilateral VCP was more often caused by thyroid surgery, while unilateral VCP was more often caused by other surgery, like carotid endarterectomy, anterior approaches to the cervical spine, and heart or great vessel surgery. Unilateral VCP was idiopathic in almost 20 % of cases. Malignancy outside the larynx was the third most common cause of unilateral VCP, being responsible for 14 % of cases. Traumatic injury causes about 6 % of all unilateral VCPs and is most frequently intubation related. Less common causes were central nervous system disease, infection, inflammation, radiation therapy, and aortic aneurysm.

Clinically, vocal cord function can be assessed by laryngoscopy, during which a stroboscopic light can confirm the absence of movement of the affected side. Symptoms of VCP include: hoarseness, vocal fatigue, loss of vocal pitch, shortness of breath and aspiration [2]. However, about 30–40 % of patients with unilateral VCP are asymptomatic [3, 4]. In these patients, presence of VCP is an incidental finding and the radiologist may be the first to report it. Due to the wide range of possible locations for lesions that can cause cord paralysis, it may be a first sign of extensive and severe pathology. Radiologists must therefore be able to recognise the imaging findings of VCP.

This review focuses on the anatomy and imaging evaluation of the vagal and recurrent laryngeal nerves and thereby the possible sites for pathology causing VCP. The imaging characteristics and imaging mimics of VCP are discussed and cases from daily practice illustrating various causes of VCP are presented.

## Anatomy of the vocal cords and their innervation by the vagal nerves and recurrent laryngeal nerves [5, 6]

**Fig. 1:**
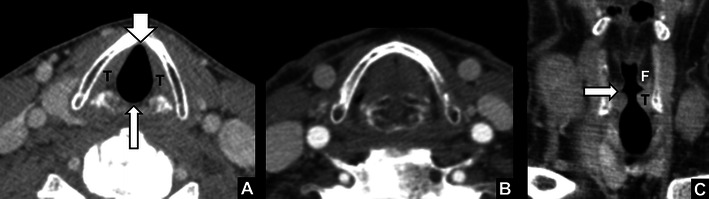
*Normal appearance of the vocal cords.* **a** Axial CT images during quiet breathing showing the anterior commissure (*short arrow*), the posterior commissure (*long arrow*) and the true vocal cords (**T**). **b** Image at the same level during breath-hold. **c** Normal appearance of the laryngeal ventricles on coronal reformat: right laryngeal ventricle (*arrow*); left true vocal cord (**T**); left false vocal cord (**F**)

The vocal cords are located in a subsite of the larynx, called the glottis. The glottis includes the true vocal cords, the anterior commissure and the posterior commissure. From medial to lateral the vocal cords consist of the mucosal surface, the vocal ligaments and the intrinsic laryngeal muscles (vocalis muscle and thyroarytenoid muscle). The anterior commissure is the midline area where the cords meet anteriorly and where they are attached to the thyroid cartilage. The posterior commissure is the mucosal surface anterior to the cricoid cartilage in between the arytenoid cartilages. Posteriorly, the vocal cords are attached to the arytenoid cartilages and laterally to the inside surface of the thyroid lamina. The medial margins are free to permit the opening and closing of the airway. During quiet respiration the cords are in a relaxed, abducted state (Fig. 1a). Breath-holding brings the cords together in an adducted midline position (Fig. 1b). Directly cranial to the vocal cords, the slit-like opening of the laryngeal ventricles separates the true vocal cords inferiorly from the false vocal cords superiorly (Fig. 1c). The movement of the vocal cords is controlled by the intrinsic laryngeal muscles. All intrinsic laryngeal muscles except the cricothyroid muscle are innervated by the recurrent laryngeal nerves, which are branches of the vagal nerves.

**Fig. 2:**
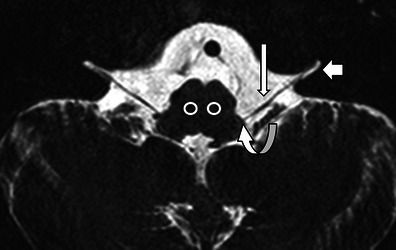
*The vagal nerves in the posterior fossa.*Axial heavily T2-weighted thin-slice MR image through the brainstem with the position of the nucleus ambiguus (*white circles*), the exit of the vagal nerves from the olivary sulcus (*curved arrow*), the cisternal segment of the vagal nerves (*long arrow*), and the pars nervosa of the jugular foramen (*short arrow*)

**Fig. 3:**
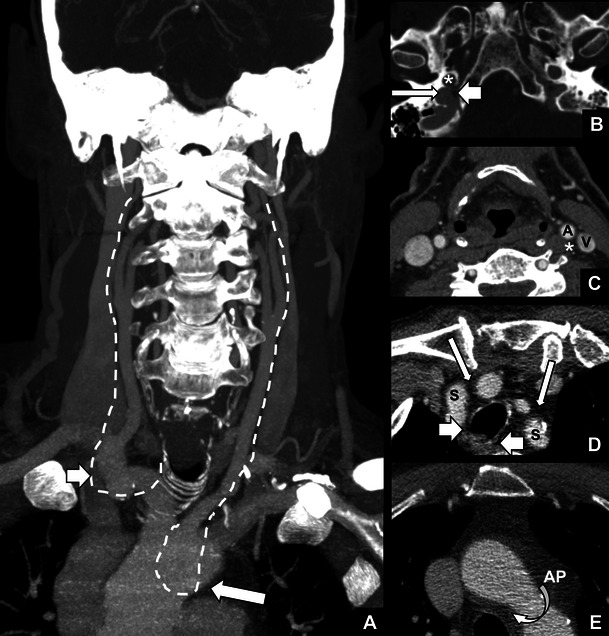
*The course of the vagal nerves and recurrent laryngeal nerves.* **a** Coronal MIP reformat showing the expected course of the vagal nerves bilaterally within the carotid sheath and proximal parts of the recurrent laryngeal nerves (*dotted lines*). The right recurrent laryngeal nerve branches just caudally to the right subclavian artery (*short arrow*) and crosses the right subclavian artery towards the tracheo-oesophageal groove. The left recurrent laryngeal nerve (*long arrow*) runs below the aortic arch. **b** Contrast enhanced CT of the skull base showing the jugular foramen with the jugular vein (*long arrow*), the pars nervosa (*short arrow*) and the internal carotid artery (*). **c** The carotid sheath with the internal carotid artery (**A**), the jugular vein (**V**) and the expected position of vagal nerve (*). **d** At the level of the subclavian arteries (**S**) the vagal nerves (*long arrows*) lie anteriorly in the upper mediastinum. At this level, the recurrent laryngeal nerves run more posteriorly in the tracheo-oesophageal groove (*short arrows*). **e** The left recurrent laryngeal nerve crosses the aortic arch from anterior to posterior (*curved arrow*) at the level of the aorto-pulmonary window (**AP**)

The vagal nerve (cranial nerve X) exits bilaterally from the medulla oblongata just lateral to the oliva through the olivary sulcus. There are three nuclei within the medulla that receive and transmit information from and to the vagal nerve. The nucleus ambiguus gives rise to motor fibres to the larynx and is located just dorsally to the inferior olive, lateral and ventral to the lower part of the fourth ventricle (Fig. 2). After exiting the medulla, the vagal nerve courses through the pre-medullary cisterns (Fig. 2) towards the pars vascularis of the jugular foramen (Fig. 3b). The nerve exits the skull through the pars vascularis of the jugular foramen and then runs within the carotid sheath together with the internal carotid artery, which lies medially to the nerve, and the jugular vein, which lies laterally (Fig. 3a and c). Upon entering the upper mediastinum the right vagal nerve crosses the right subclavian artery ventrally and then courses medially and dorsally toward the right side of the trachea. Just caudally to the right subclavian artery, the right recurrent laryngeal nerve branches from the vagal nerve (Fig. 3a and e). The right recurrent laryngeal nerve then runs dorsally to the right subclavian artery in a cranial and medial direction to reach the larynx via the right trachea-oesophageal (TE) groove (Fig. 3d). The left vagal nerve lies ventrally to the left subclavian artery upon entering the upper mediastinum (Fig. 3a and d). It remains on top of the subclavian artery and runs over the aortic arch on the left side (Fig. 3a). The left recurrent laryngeal nerve branches from the vagal nerve just below the ligamentum arteriosum at the level of the aorto-pulmonary (AP) window and moves dorsally under the aortic arch in the direction of the left TE groove (Fig. 3a and e). From there, like the recurrent laryngeal nerve on the right, it moves upward to the larynx. Both recurrent laryngeal nerves enter the larynx through the inferior constrictor muscles at the level of the cricothyroid joint. Since the left recurrent laryngeal nerve is longer than the right nerve (12 cm versus 6 cm), left VCP is more commonly encountered [7].

## Imaging strategy

### Brainstem and skull base

Bilateral VCP is indicative of a central cause in the medulla oblongata [8]. Acute onset of symptoms also points toward a central cause. The area of the medullary nuclei of the vagal nerve can best be evaluated with magnetic resonance imaging (MRI) [9, 10], using T2-weighted imaging, diffusion-weighted imaging (DWI) and T1-unenhanced and gadolinium-enhanced imaging. Possible pathology in the medulla oblongata comprises demyelinating diseases, infarction and intra-axial tumours [1, 7, 11, 12]. The cisternal part is best visualised by high-resolution heavily T2-weighted sequences and T1-weighted imaging with gadolinium [13, 14]. At this location, the vagal nerve can be affected by external compression by extra-axial masses, vascular structures, or by pathology that affects the nerve itself like schwannoma, paraganglioma or neuritis [1, 7, 11, 12]. If multiple cranial nerves are involved, the jugular foramen should be scrutinised. Pathology in and around the jugular foramen is best depicted by an MRI of the posterior fossa comprising T2-weighted imaging, DWI and T1-unenhanced and gadolinium-enhanced imaging [9, 13, 14].

### Extracranial vagal nerves and recurrent laryngeal nerves

To depict pathology in the course of the extracranial vagal nerves and the recurrent laryngeal nerves, we prefer contrast-enhanced computed tomography (CT) from the midbrain to the aortic arch including the AP window (Fig. 3a) [15]. With multi-slice detector CT scanners the whole volume can be acquired with isotropic voxels with a slice thickness of 0.625 mm. The scan duration is only several seconds, which allows for a minimum of image quality degradation by breathing-induced and swallowing-induced motion artefacts. The CT should be acquired during quiet breathing, in order to bring the vocal cords to an abducted position (Fig. 1a). The isotropic voxels make it possible to make reconstructions in any plane of choice (preferred slice thickness of 2–3 mm). Axial reconstructions should always be obtained parallel to the true vocal cords for optimal diagnostic accuracy. Frequently occurring pathologies that cause extracranial vagal or recurrent laryngeal nerve palsy are lung cancer with mediastinal lymph node metastases and squamous cell carcinoma of the neck with or without local lymph node metastases [1, 7]. However, other pathologies like infectious processes, benign masses or malignancies of other structures in the neck and upper mediastinum can all cause VCP [1, 7, 11, 12].

## Imaging characteristics of VCP

**Fig. 4:**
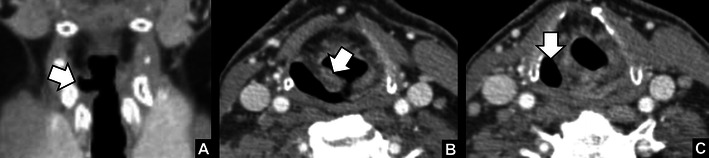
*Specific imaging characteristics of vocal cord paralysis (right side).* **a** Widening of the right laryngeal ventricle (*arrow*). **b** Medial deviation and thickening of the right aryepiglottic fold (*arrow*). **c** Dilatation of the right piriform sinus (*arrow*)

**Fig. 5:**
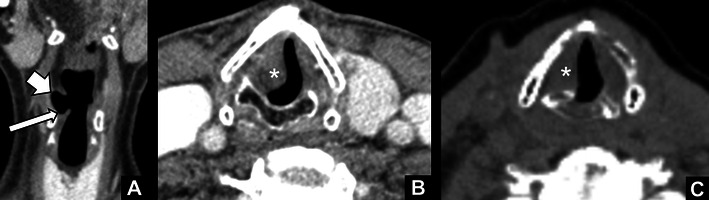
*Supportive imaging characteristics of vocal cord paralysis (VCP).* **a** Pointed right vocal cord (*long arrow*) on coronal reformat due to atrophy; note the widening of the ipsilateral laryngeal ventricle (*short arrow*). **b** Subglottic fullness due to sagging of the paralysed right vocal cord (*). **c** Paramedian position of the paralysed right vocal cord (*)

The most specific findings of unilateral VCP are [15, 16]: (1) widening of the laryngeal ventricle (Fig. 4a), (2) medial deviation and thickening of the aryepiglottic fold (Fig. 4b), and (3) dilatation of the piriform sinus (Fig. 4c). The widening of the laryngeal ventricle is the passive result of atrophy of the ipsilateral cord. The changes in the aryepiglottic fold and the piriform sinus are a result of paralysis of the posterior cricoarytenoid muscle. This muscle is the only muscle that abducts the vocal cords. Atrophy of this muscle and the inability to abduct the cord results in anteromedial rotation of the arytenoid, medial deviation of the aryepiglottic fold, and (passive) dilatation of the piriform sinus. In addition to these imaging findings, several other less specific supportive signs have been described in the literature [17]. On coronal reformats, the atrophied cord can have a pointed appearance due to reduced bulk (Fig. 5a). On axial images, the subglottic area on the affected side can appear full due to sagging of the paralysed cord (Fig. 5b). Also the paralysed cord often has a paramedian position due to the inability to abduct (Fig. 5c). Findings that can help to localise the level of vagal nerve impairment are involvement of other cranial nerves. For example, pathology at the level of the jugular foramen will frequently result in involvement of the glossopharyngeal nerve (IX), accessory nerve (XI), and hypoglossal nerve (XII), leading to atrophy of the pharyngeal constrictor muscles (IX), sternocleidomastoid and trapezius muscles (XI), and tongue muscles (XII) respectively.

## Mimics of VCP

**Fig. 6:**
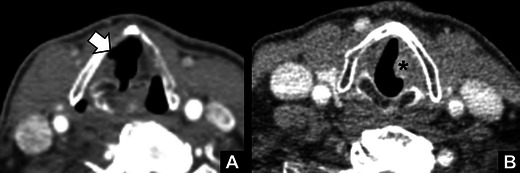
*Mimics of VCP.* **a** Tilted axial reformat through the larynx showing subglottic air (*arrow*) projecting anterior to the angulated normal right true vocal cord, mimicking a widened laryngeal ventricle seen in VCP. **b** Small squamous cell carcinoma of the left vocal cord (*) mimicking the paramedian position seen in VCP

**Fig. 7:**
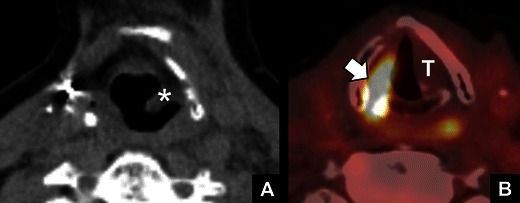
*FDG-PET findings in VCP.* **a** Non-contrast CT image showing medial deviation and thickening of the left aryepiglottic fold (*) indicative of left VCP. **b** Fused PET-CT showing FDG uptake in the right vocal cord (*arrow*) due to compensatory hypertrophy. This should not be confused with disease. Note the paramedian position of the left true vocal cord (**T**)

**Fig. 8:**
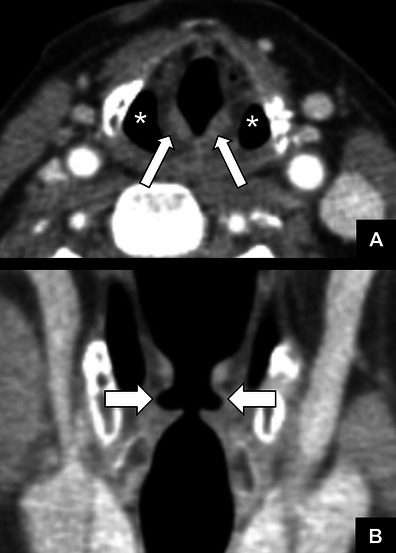
*Bilateral VCP.* **a** Axial CT reformat showing bilateral medial deviation and thickening of the aryepiglottic fold (*arrows*) and bilateral dilatation of the piriform sinuses (*). **b** Coronal CT reformat showing bilateral widening of the laryngeal ventricles (*arrows*)

Misdiagnosis of VCP may result in unnecessary additional evaluation or inappropriate management and delay. To avoid misdiagnosis, the radiologist must be aware of several mimics of VCP. First of all, the axial CT reformats have to be angulated parallel to the true vocal cords with both crycoarytenoid joints within the same plane. Tilted axial reformats may lead to subglottic air projecting anterior to the angulated normal vocal cord. This imaging appearance may mimic a widened laryngeal ventricle and thereby VCP (Fig. 6a). Second, malignant infiltration of the vocal cord can result in immobilisation or thickening, mimicking a paramedian position of the cord (Fig. 6b). However, this may only be relevant in incidental detection of a unilaterally abnormal vocal cord. When patients present with symptoms of VCP, laryngoscopy is usually performed prior to CT imaging. During laryngoscopy, focal lesions of the vocal cord can be easily differentiated from VCP. Third, traumatic injury to the arytenoid cartilage with medial dislocation may mimic VCP [18]. This dislocation can be either the result of direct blunt trauma to the larynx or intubation. Especially after intubation the differentiation between traumatic dislocation and paralysis can be difficult since intubation can cause both. VCP paralysis can be the result of compression of the anterior motor branch of the recurrent laryngeal nerve between the inflated cuff of the endotracheal tube, the lateral projection of the abducted arytenoid, and the thyroid cartilage [19]. In addition, intubation can result in fibrosis of the vocal cord, which can also mimic VCP. A confusing imaging finding, which the radiologist should be aware of, can be seen on ^18^F-fluorodeoxyglucose positron emission tomography (FDG-PET) exams of patients with VCP. In such cases, the PET may show increased metabolism in the unaffected vocal cord due to compensatory hypertrophy (Fig. 7b). This finding mimics malignancy on the unaffected side [20]. Usually, correlation of the PET findings with the co-registered CT images will show signs of VCP on the non-metabolic side. Another confusing finding is bilateral VCP. The typical imaging characteristics of unilateral VCP can in these cases occur on both sides, making them more difficult to identify (Fig. 8).

## Cases

**Fig. 9:**
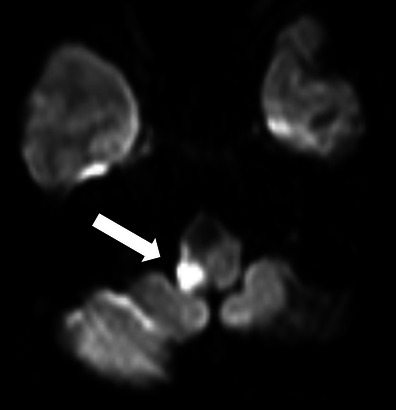
*Brainstem infarction.*A 55-year-old woman presenting with VCP after endovascular coiling of a right vertebral artery aneurysm. DWI at the level of the medulla oblongata showed a small area of restricted diffusion directly at the site of the right vagal nerve nucleus, indicative of infarction (*arrow*)

**Fig. 10:**
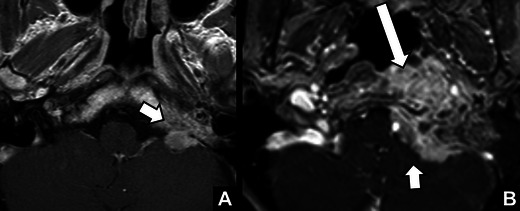
*Skull base metastasis.*A 66-year-old man with a history of oropharynx carcinoma treated with radiation therapy 1 year earlier presenting with left VCP. FDG-PET revealed a hypermetabolic lesion in the left skull base presumed to be a metastasis. **a** Axial T1-weighted contrast-enhanced images at the level of the skull base showed a heterogeneously enhancing mass in the left cerebellomedullary cistern (*arrow*). The mass shows infiltrative growth into the skull base and jugular foramen, and thereby the vagal nerve, causing left VCP. The imaging findings were suspicious for metastatic disease. **b** Follow-up T1-weighted contrast-enhanced images with fat saturation showed progression of the skull-base mass with infiltrative growth into the nasopharynx (*long arrow*) and mass effect on the cerebellum and pons (*short arrow*)

**Fig. 11:**
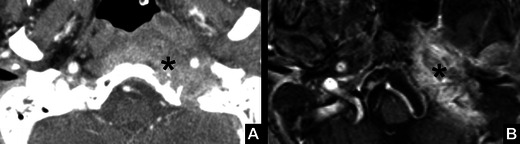
*Osteomyelitis of the skull base.* A 74-year-old man presenting with slowly progressive symptoms of left hypoglossal nerve palsy and left VCP. CT and MR images at the level of the skull base (*not displayed*) showed fluid in the mastoid and middle ear bilaterally. **a** Contrast-enhanced CT and **b**) T1-weighted contrast-enhanced images with fat saturation showed infiltration of the area between the petrous apex and the temporo-mandibular joint by a destructive enhancing mass (*). The imaging findings suggested osteomyelitis and soft tissue infection. The jugular foramen and thereby the vagal nerve were obviously involved, causing VCP. The patient underwent left-sided exploration and mastoidectomy revealing massive infection of the middle ear, skull-base and neck

**Fig. 12:**
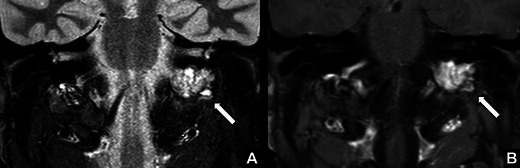
*Glomus jugulare tumour.*A 42-year-old man presenting with slowly progressive symptoms of left VCP with no other apparent laryngeal pathology. **a** Coronal T2-weighted MRI images and **b** T1-weighted contrast-enhanced fat-saturated images showed a T2 hyperintense avidly enhancing, smooth lobulated mass, with a salt and pepper appearance at the left jugular foramen (*arrows*) typical of glomus jugulare tumour

**Fig. 13:**
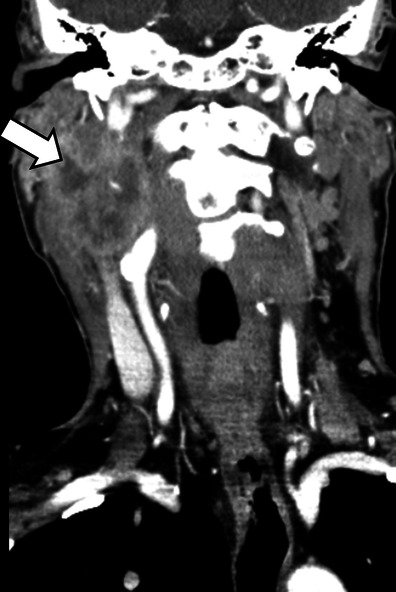
*Lymph node metastasis in the neck from oropharyngeal squamous cell carcinoma.*A 66-year-old man with a history of smoking and alcohol abuse presenting with a swelling on the right side of the neck and right VCP. Coronal contrast enhanced CT images showed a large lymph node conglomerate at level IIb (*arrow*) on the right side of the neck, with necrosis, extra nodal spread and extensive involvement of the right carotid space explaining the right VCP in this patient

**Fig. 14:**
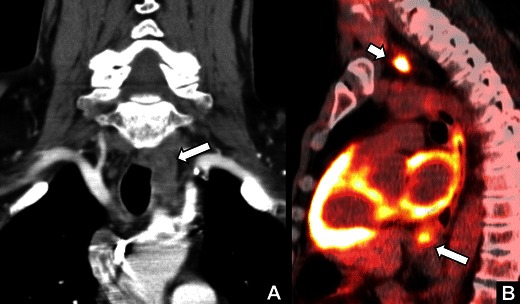
*Paratracheal lymph node metastasis from oesophageal carcinoma.* A 53-year-old man with a history of heavy smoking and drinking presenting with left VCP. **a** Coronal contrast-enhanced CT images showed a mass in the left TE groove in the upper mediastinum in the course of the left recurrent laryngeal nerve (*arrow*). **b** Sagittal fused FDG-PET-CT images showed avid FDG uptake in the mass (*short arrow*) and in a thickened portion of oesophagus just below the carina (*long arrow*). The patient was diagnosed with oesophageal carcinoma with left paratracheal lymph node metastasis causing left sided VCP

Figures 9, 10, 11, 12, 13, 14 15 and 16 show illustrative cases from daily practice with various benign and malignant causes of VCP.

**Fig. 15:**
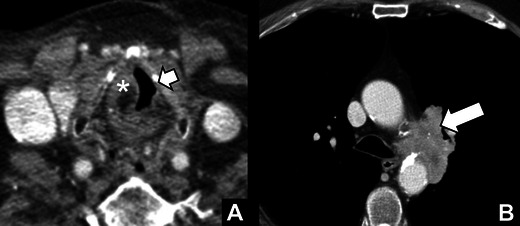
*Lung cancer with involvement of the aorto-pulmonary (AP) window.*72 year old male smoker with a history of squamous cell carcinoma of the left ear presenting with functional dysphonia caused by left VCP. **a** Axial contrast-enhanced CT of the larynx showing complete atrophy of the left true vocal cord (*short arrow*) and hypertrophy of the right true vocal cord (*). **b** Images at the level of the AP window showed a large supra-hilar mass in the left lung infiltrating the AP-window (*arrow*), thereby explaining left VCP in this patient. Biopsy revealed a non-small cell lung carcinoma

**Fig. 16:**
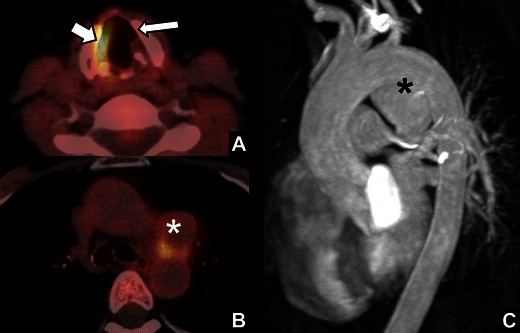
*Aortic arch aneurysm.* A 44-year-old patient with a history of systemic lupus erythematosus and herpes zoster infection presenting with symptoms of left VCP. Because of the patient’s history, a FDG-PET with low-dose CT was obtained. **a** Fused PET-CT image at the level of the cricoid showed widening of the left laryngeal ventricle (*long arrow*), note the increased FDG-uptake in the unaffected right vocal cord due to hypertrophy (*short arrow*). **b** Images at the level of the aortic arch showed a mass in the AP-window without FDG uptake (*). **c** Contrast-enhanced MR angiography of the aortic arch showed a saccular aneurysm (*) causing compression of the vagal nerve at the site of the branching of the left laryngeal recurrent nerve, thereby causing VCP

## Conclusions

VCP can be caused by any offending lesion in the course of the vagal and recurrent laryngeal nerves, between the medulla oblongata and the aortic arch. Since VCP may be the first presentation of pathology the radiologist has to be aware of (1) the imaging characteristics and mimics of VCP, (2) the expected course of the vagal nerves and recurrent laryngeal nerves, and (3) the different types of pathology that can occur along their course.
